# Correction: Hip thrust and back squat training elicit similar gluteus muscle hypertrophy and transfer similarly to the deadlift

**DOI:** 10.3389/fphys.2026.1987823

**Published:** 2026-10-28

**Authors:** Daniel L. Plotkin, Merlina A. Rodas, Andrew D. Vigotsky, Mason C. McIntosh, Emma Breeze, Rachel Ubrik, Cole Robitzsch, Anthony Agyin-Birikorang, Madison L. Mattingly, J. Max Michel, Nicholas J. Kontos, Sarah Lennon, Andrew D. Frugé, Christopher M. Wilburn, Wendi H. Weimar, Adil Bashir, Ronald J. Beyers, Menno Henselmans, Bret M. Contreras, Michael D. Roberts

**Affiliations:** 1 School of Kinesiology, Auburn University, Auburn, AL, United States; 2 Department of Psychological Sciences, Auburn, AL, United States; 3 Departments of Biomedical Engineering and Statistics, Evanston, IL, United States; 4 Department of Neuroscience, Northwestern University, Chicago, IL, United States; 5 College of Nursing, Auburn University, Auburn, AL, United States; 6 MRI Research Center, Auburn University, Auburn, AL, United States; 7 International Scientific Research Foundation for Fitness and Nutrition, Amsterdam, Netherlands; 8 BC Strength, San Diego, CA, United States

**Keywords:** hip thrust, back squat, gluteus maximus, strength, hypertrophy

In the originally published article, the **Funding** statement read as follows:

“Funding for this study was made possible through gift funds (some of which were donated by the International Scientific Research Foundation for Fitness and Nutrition and BC) to the MR laboratory. Other financial sources included indirect cost sharing (generated from various unrelated contracts) from the School of Kinesiology, MM being fully supported through a T32 NIH grant (T32GM141739), and DP being fully supported by a Presidential Graduate Research Fellowship (fund cost-sharing from Auburn University’s President’s office, the College of Education, and the School of Kinesiology).”

However, the co-corresponding author MDR did not originally acknowledge an in-kind equipment donation to his laboratory. The corrected **Funding** statement with equipment donation appears below:

“Funding for this study was made possible through gift funds (some of which were donated by the International Scientific Research Foundation for Fitness and Nutrition and BC) to the MR laboratory. BC Strength also donated the Thruster 3.0 apparatus used in the study. Other financial sources included indirect cost sharing (generated from various unrelated contracts) from the School of Kinesiology, MM being fully supported through a T32 NIH grant (T32GM141739), and DP being fully supported by a Presidential Graduate Research Fellowship (fund cost-sharing from Auburn University’s President’s office, the College of Education, and the School of Kinesiology).”

In the originally published article, the **Conflicts of interest** statement read as follows:

“BC and MH disclose that they sell exercise-related products and services. However, neither was involved in any aspect of the study beyond assisting with the study design and providing funds to partially cover participant and MRI costs through a gift to the laboratory of MR. The results of the study are presented clearly, honestly, and without fabrication, falsification, or inappropriate data manipulation. The remaining authors declare that the research was conducted in the absence of any commercial or financial relationships that could be construed as a potential conflict of interest.”

In the published article, the **Conflicts of interest** statement has been updated by co-corresponding author MDR to provide additional clarity regarding off-site collaborator and donor involvement. The updated **Conflicts of interest** statement appears below:

“BC (via BC Strength) and MH disclose that they sell exercise-related products and services. Neither was involved in participant recruitment, training, data collection, or data analysis; their involvement was limited to assisting with the study design. The results of the study are presented clearly, honestly, and without fabrication, falsification, or inappropriate data manipulation. The remaining authors declare that the research was conducted in the absence of any commercial or financial relationships that could be construed as a potential conflict of interest.”

In the published article, this study was not prospectively registered at the time of study initiation (2022). The study has since been registered by co-corresponding author MDR at ClinicalTrials.gov (registration #: NCT7760220; registration date: 8-11-2026). This has no bearing on the originally reported data or results of the study.

The **Publisher’s note** has been updated to include this notice:

“Frontiers’ editorial policy, in line with ICMJE guidance, requires clinical trials to be registered prospectively, before participant enrolment.

Following assessment, the journal determined that the absence of prospective registration in this case did not provide grounds to conclude that the article’s findings are unreliable or that the delay was intended to enable biased reporting. This determination was based on the available ethics documentation, the authors’ explanation, and the absence of evidence that the study design or reported outcomes were changed after data collection began.

The study has since been registered retrospectively, and the registration details are provided above. The journal has permitted retrospective registration as a case-specific exception.

All claims expressed in this article are solely those of the authors and do not necessarily represent those of their affiliated organizations, or those of the publisher, the editors and the reviewers. Any product that may be evaluated in this article, or claim that may be made by its manufacturer, is not guaranteed or endorsed by the publisher.”

The original version of this article has been corrected.

